# Meetings of Societies

**Published:** 1940

**Authors:** 


### Meetings of Societies

### Bristol Medico-Chirurgical Society.

ONE afternoon session was held on 7th November, when Major C. S. Marriott, M.D., F.R.C.P., and Captain Kekwik, M.B., M.R.C.P., of the Army Blood Transfusion Service spoke on the "Uses and Abuses of Blood, Plasma, and Intravenous Salines."

It is hoped occasionally to hold afternoon meetings during the coming session.

